# Rapid Assessment of Sulfate Resistance in Mortar and Concrete

**DOI:** 10.3390/ma17194678

**Published:** 2024-09-24

**Authors:** Seyedsaleh Mousavinezhad, William K. Toledo, Craig M. Newtson, Federico Aguayo

**Affiliations:** 1 Department of Civil Engineering, New Mexico State University, Las Cruces, NM 88003, USA; 2 Sandia National Laboratories, Albuquerque, NM 87123, USA; 3 Department of Construction Management, University of Washington, Seattle, WA 98195, USA

**Keywords:** accelerated test method, concrete, metakaolin, mortar, natural pozzolan, sulfate attack

## Abstract

Extensive research has been conducted on the sulfate attack of concrete structures; however, the need to adopt the use of more sustainable materials is driving a need for a quicker test method to assess sulfate resistance. This work presents accelerated methods that can reduce the time required for assessing the sulfate resistance of mixtures by 70%. Class F fly ash has historically been used in concrete mixtures to improve sulfate resistance. However, environmental considerations and the evolving energy industry have decreased its availability, requiring the identification of economically viable and environmentally friendly alternatives to fly ash. Another challenge in addressing sulfate attack durability issues in concrete is that the standard sulfate attack test (ASTM C1012) is time-consuming and designed for only standard mortars (not concrete mixtures). To expedite the testing process, accelerated testing methods for both mortar and concrete mixtures were adopted from previous work to further the development of the accelerated tests and to assess the feasibility of testing the sulfate resistance of mortar and concrete mixtures rapidly. This study also established criteria for interpreting sulfate resistance for each of the test methods used in this work. A total of 14 mortar mixtures and four concrete mixtures using two types of Portland cement (Type I and Type I/II) and various supplementary cementitious materials (SCMs) were evaluated in this study. The accelerated testing methods significantly reduced the evaluation time from 12 months to 21 days for mortar mixtures and from 6 months to 56 days for concrete mixtures. The proposed interpretation method for mortar accelerated test results showed acceptable consistency with the ACI 318-19 interpretations for ASTM C1012 results. The interpretation methods proposed for the two concrete sulfate attack tests demonstrated excellent consistency with the ASTM C1012 results from mortar mixtures with the same cementitious materials combinations. Metakaolin was shown to improve sulfate resistance for both mortar and concrete mixtures, while silica fume and natural pozzolan had a limited impact. Using 15% metakaolin in mortar or concrete mixtures with Type I/II cement provided the best sulfate resistance.

## 1. Introduction

Concrete is one of the most widely used building materials globally, with nearly 10 billion cubic meters utilized annually [1]. However, as concrete structures age, many show signs of premature deterioration. Among the various durability issues in cement-based materials, sulfate attack is a significant concern for concrete structures such as dams, bridge piers, pavement, and foundations such as footings and piles [2,3,4,5], leading to substantial maintenance costs—part of the over USD 4 trillion annual global expense related to corrosion [6,7]. The degradation of reinforced concrete structures caused by sulfate attack from external sources, such as groundwater, has been studied by many researchers across the world [8,9,10,11,12]. It has been found that several factors such as pH, temperature, humidity, and sulfate concentration can influence the severity and type of external sulfate attack, resulting in varying effects on concrete, including loss of paste–aggregate bonding, softening, expansion, and associated cracking and surface spalling [13].

Generally, sulfate ions from external sources penetrate the concrete matrix, changing the pore solution equilibrium and resulting in the formation of expansive compounds. Various theories have been proposed to explain how sulfate attack causes expansion and damage in concrete. These include mechanisms such as topochemical reactions occurring at solid–liquid interfaces [14], pressure generated by crystallization [15], material swelling [16], and an increase in the volume of solid products [17]. For instance, research by Müllauer et al. [18] suggests that the formation of ettringite within small pores (10–50 nm) creates internal stresses as high as 8 MPa, which can surpass the tensile strength of the concrete matrix. Additionally, the expansion of solids due to sulfate attack can cause the solid-phase volume of sulfate products to increase by up to 24% [19].

There are generally two types of external sulfate attack, physical and chemical attacks [20]. Physical attack causes cracking and erosion of the concrete surface due to phase changes in the sulfate solution [21]. A typical indicator of this type of attack is the presence of sodium sulfate deposits, or “bloom”, on exposed surfaces. While this may seem like a surface-level issue, it often indicates deeper chemical and microstructural damage within the concrete [22]. Chemical attack leads to cracking, increased permeability, and reduced strength due to volumetric expansion in hardened concrete caused by changes in the hydration products within the cement paste [23,24,25]. Two principal processes of chemical sulphate attack include the formation of ettringite and gypsum [26]. The primary focus of this study is on chemical sulfate attack, which is a complex phenomenon that is not yet fully understood.

To address various durability issues in concrete, improving its microstructure is crucial [27]. Specifically, to mitigate sulfate attack, using appropriate combinations of cement and supplementary cementitious materials (SCMs) with a low water-to-cementitious materials ratio (w/cm) is essential [28,29,30,31,32]. SCMs rich in active SiO_2_ or active Al_2_O_3_ are generally known to improve sulfate resistance by decreasing permeability [33,34,35], which limits the ingress into and movement of external sulfate ions in concrete. SCMs also consume reactive tricalcium aluminate (C_3_A) compounds in the matrix, which decreases the formation of aluminate hydrates available to react with sulfates provided by external sources [36,37]. Additionally, SCMs increase the sulfate resistance by decreasing Ca(OH)_2_ content through the pozzolanic reaction and forming secondary calcium silicate hydrate (CSH), which creates a coating on alumina-rich phases to hinder the formation of secondary ettringite [36,38,39]. For instance, class F fly ash, a byproduct of coal combustion in electricity production, has historically been used to improve the sulfate resistance and other durability and mechanical properties of concrete, and it also reduces materials costs and improves sustainability [40]. Baloch et al.’s research [41] also indicates that incorporating low-calcium fly ash and slag positively affects the residual mechanical properties of fiber-reinforced concrete with long-term exposure to sodium sulfate solutions.

Unfortunately for the concrete industry, class F fly ash has become difficult to procure due to environmental concerns and changes in the energy industry. Therefore, it has become necessary to identify alternative SCMs capable of mitigating sulfate attack while being cost-effective and environmentally friendly. Another challenge in addressing sulfate-related issues in concrete is that the existing sulfate attack test standards in the US have limitations and discrepancies. The most commonly used standard, ASTM C1012 [42], only evaluates mortar mixtures, not concrete mixtures. It also requires a long testing period of up to 18 months to obtain reasonable results, especially when SCMs are used, even with aggressive sulfate concentrations. Additionally, ASTM C1012 [42] may not accurately replicate real-world performance, and reproducibility across different laboratories is often poor. This standard only allows the use of sodium sulfate and magnesium sulfate solutions, disregarding other types of sulfate present in real-world conditions.

This study focuses primarily on the development of accelerated tests that can reduce the time required for assessing the sulfate resistance of mixtures by 70%. Although various standards for sulfate attack testing exist, each has fundamental limitations, such as extensive testing durations and limited applicability across various types of cement-based materials (similar to the limitations of ASTM C1012). The tests developed in this research provide more rapid and reliable sulfate attack testing methods, and their suitability for both mortar and concrete mixtures is established. To achieve this, accelerated testing methods were developed for both mortar and concrete mixtures, reducing the testing time for sulfate resistance evaluation, and a method derived from ASTM C1012 was employed to assess sulfate resistance in concrete mixtures. Interpretation methods were then proposed for each of these three approaches, the accelerated testing method for mortar, the accelerated testing method for concrete, and the ASTM-based testing method for concrete, increasing their practicality. To produce a diverse range of mortar and concrete mixtures containing various SCMs, 14 mortar mixtures and 4 concrete mixtures, using two types of Portland cement (Type I and Type I/II) and various SCMs (fly ash, silica fume, metakaolin, a locally available natural pozzolan, and combinations of these SCMs), were designed and evaluated. To analyze the reliability and repeatability of the results from the different test methods, the coefficient of variation (CoV) values for the accelerated mortar and concrete tests were compared to the CoV values from ASTM C1012 testing.

## 2. Literature Review

This section provides a literature review for sulfate attack mechanisms, sulfate attack mitigation methods, and current sulfate attack test methods.

### 2.1. Sulfate Attack

There are two types of sulfate attack in cementitious materials: internal sulfate attack originating from excess sulfate in the original concrete mixture and external sulfate attack caused by sulfate ingress through the surrounding environment such as soil and seawater [43,44]. External sulfate attack has two facets, chemical and physical, leading to issues in concrete such as reduced strength, cracking, and surface scaling, with potential concrete failure in as little as five years [26].

Physical sulfate attack results in cracking and erosion of the concrete surface due to phase changes (crystallization and cyclic hydration–dehydration) from sulfate penetration generating internal stresses [21]. Chemical sulfate attack is characterized by sulfate sources altering hydration products, causing volumetric expansion that leads to cracking, increased permeability, and strength loss [23,24,25]. It is important to note that soluble sulfate attack can induce both physical and chemical damage [20]. This study focuses on chemical sulfate attack, a less understood form of concrete deterioration that is subject to debates about its mechanisms and the correlation between laboratory tests and field performance [2,3,4,45].

### 2.2. Ettringite, Gypsum, and Thaumasite Formation

Ettringite, gypsum, and thaumasite can form when sulfates react with cement paste compounds such as monosulfate, portlandite, and CSH [2,4,5,46,47,48,49]. These three products are the primary contributors to concrete deterioration following sulfate attack.

Ettringite is an expansive product that forms when monosulfate hydrate and calcium aluminate hydrate react with sulfate in the presence of calcium hydroxide (CH) and water. The expansion of ettringite can occur due to factors such as an increase in solid volume [17], swelling [16,50], topochemical reaction [14,51], or crystal growth [15,52]. Another detrimental product of sulfate attack, gypsum, is typically found near the concrete surface and results from cation exchange between CH and sulfate. Generally, gypsum formation results in a reduction in pH, stiffness, and strength; however, the mechanisms underlying the detrimental effects of gypsum remain debatable. Some studies suggest that gypsum formation leads to expansion [53], while others indicate the opposite [54]. The third product of sulfate attack, thaumasite, forms when silicate and carbonate react with external sulfate in the presence of water, leading to softening, expansion, and concrete cracking. In contrast to ettringite and gypsum, thaumasite consumes CSH, potentially leading to disintegration of the cement matrix, causing more severe concrete damage than ettringite or gypsum formation [55,56].

### 2.3. Mitigation of Sulfate Attack

The extent and rate of sulfate attack deterioration depend on several factors, including cement type, the w/cm, cation and sulfate concentrations, mineral admixtures, and exposure temperature [57]. Mitigating sulfate attack typically involves three primary strategies: reducing w/cm to reduce concrete permeability, limiting C_3_A content in cement, and decreasing CH content in the matrix [58,59,60].

Calcium aluminate hydrate and CH are main contributors to sulfate attack reactions. Methods that limit these elements in concrete can improve sulfate resistance. While ASTM C150 [61] limits C_3_A content in Type II and Type V cements to create sulfate-resistant cements, this measure alone might not suffice, since CH continues to play a crucial role. Partially replacing Type II and V cements with SCMs further improves sulfate resistance. This is achieved by reducing C_3_A content (due to using less cement) and by consuming CH through the pozzolanic reactivity of SCMs. This limits the availability of aluminate hydrates and CH to react with sulfate. SCMs also improve the pore structure and increase density, making concrete less susceptible to external sulfate ingress [36]. Several studies have shown the effectiveness of partially replacing cement with SCMs such as fly ash [62,63], metakaolin [64,65], silica fume [35,66], and ground granulated blast-furnace slag [67,68] in improving concrete sulfate resistance.

### 2.4. Sulfate Attack Test Methods

There are different standardized methods for assessing cementitious materials subjected to external sulfate attack, each with some limitations, necessitating further research for more accurate assessments. ASTM C452-21 [69] involves adding gypsum to Portland cement prior to mixing, which accelerates internal ettringite formation, but it is not applicable for blended cements and does not simulate the field exposure of concrete to sulfate, which involves the ingress of sulfates into concrete. ASTM C1012 [42], a more common worldwide method, is compatible with blended cement; however, it is limited to mortar (not concrete) and is time-consuming. The United States Bureau of Reclamation (USBR) recommends a similar approach to the ASTM C1012 (USBR 4908 [70]) but with extended testing periods (one to two years) [71]. Additionally, the Canadian standard (CSA A3004-13 [72]) assesses the sulfate resistance of blended cement mortars (similar to the ASTM C1012). This method includes procedures for both ettringite and thaumasite attack, but the low-temperature test is criticized for being excessively aggressive and not representative of actual sulfate resistance.

In Europe, a unified method is lacking, but the European Committee for Standardization (CEN) has issued a technical report [73] on sulfate resistance analysis, compiling data from a wide range of sources (over 100 publications). The Swiss Standard (SN 505262-1 [74]) uses aggressive cycles of drying and immersion in a sulfate solution to accelerate sulfate ingress in a manner that does not accurately represent actual sulfate exposure. There are also several Chinese standards (such as GB/T 749-2008 [75]); however, they have limitations such as vague specifications for specimen size and quantity, a long testing time, and lack of clarity on the most suitable testing solution, optimal solution concentration, and immersion duration.

In summary, each of these standardized methods presents its advantages and challenges when assessing the sulfate resistance of cementitious materials. Researchers should carefully consider these limitations in their choice of testing methods to ensure the applicability of the results to real-world scenarios. Additionally, the selection of a testing method should be influenced by factors such as the type of cement, sulfate exposure conditions, and the specific objectives of the study.

## 3. Methodology

### 3.1. Materials

The materials used in this study consisted of locally sourced aggregates and six types of cementitious materials. The aggregates included sand and coarse aggregate that were obtained from GCC in Las Cruces, NM, USA. Table 1 and Table 2 present the physical properties and particle size distribution for these aggregates, respectively.

As previously mentioned, one of the aims of this study was to evaluate the potential of replacing fly ash, which is expected to have a limited availability in the future, with other locally available SCMs such as silica fume, metakaolin, and natural pozzolan to improve sulfate resistance. The six cementitious materials used in this study included a Type I cement from Buda, TX, USA (manufactured by Texas Lehigh Cement), a Type I/II cement produced by GCC, Las Cruces, NM, USA, a commercially available silica fume (MasterLife SF 100) produced by BASF Chemical Company in Cambridge, MA, USA, a metakaolin product (GMK-S5) manufactured by Grace in Aiken, SC, USA, a natural pozzolan (pumicite) mined near Española, NM, USA, and a class F fly ash produced at the San Juan power plant in northern New Mexico, USA (distributed by the Salt River Materials Group in Farmington, NM, USA). Table 3 presents the chemical properties, chemical composition, and physical properties for the cementitious materials used in this study.

### 3.2. Mixture Proportions

A total of 18 cementitious mixtures were produced in this study, including 14 mortar and 4 concrete mixtures. Each mixture contained either Type I or Type I/II Portland cement as the primary cementitious material. Additionally, SCMs including silica fume (SF), metakaolin (MK), pumicite (NP) as a natural pozzolan, and fly ash (FA), or a combination of these SCMs were added to some of the mixtures. SCMs were used to partially replace Portland cement in these mixtures (by mass). The proportions for these mixtures are presented in the following sub-sections.

#### 3.2.1. Mortar Mixtures

The study included 14 mortar mixtures, 9 of which used Type I and 5 of which used Type I/II Portland cement. The two control mortar mixtures (using Type I and Type I/II Portland cements) were proportioned using ASTM C1012 [42], which states that mortar mixtures without SCM should have one part cement to 2.75 parts sand (by mass) and a fixed water-to-cement ratio of 0.485. For mortar mixtures containing SCMs, ASTM C1012 [42] requires adjusting the water content of mixtures containing SCMs to achieve a flow value of the control mixture ±5%. This adjustment typically results in different w/cm ratios, which leads to greater variability in sulfate testing results since sulfate resistance is strongly influenced by the w/cm ratio [23]. Therefore, a fixed w/cm ratio of 0.485, similar to the control mixtures, was used for mixtures containing SCMs. Table 4 presents the batch quantities for the mortar mixtures.

To interpret the names of the mixtures, the first letter indicates the type of the mixture (M represents mortar and C represents concrete), the letter and number following the first hyphen indicate the type of cement used, with T1 representing Type I Portland cement and T12 representing Type I/II cement, and the letter and subsequent numbers after the second hyphen indicate the type of SCMs used in the mixtures, along with their respective concentrations. The SCM acronyms used in this study are SF for silica fume, MK for metakaolin, NP for natural pozzolan, and FA for fly ash. The numbers following the SCM type represent the ratio of the SCMs to the total mass of cementitious materials (%). For instance, M-T1-MK22.5FA7.5 indicates a mortar mixture with Type I Portland cement, containing 22.5% metakaolin and 7.5% fly ash.

#### 3.2.2. Concrete Mixtures

Four mixtures were developed to produce concrete prisms for this study that included Type I and Type I/II cements with (1) no addition of SCMs and (2) 15% cement mass replacement with metakaolin. These four mixtures were selected to provide a broader range of concrete specimens in terms of durability properties, allowing for a more comprehensive validation of the testing methods used in this study for concrete mixtures. Other studies have shown that an ideal dosage for cement replacement with metakaolin is between 10 to 20%, providing optimal performance in terms of mechanical properties, durability, and the microstructure of the concrete [76]. Therefore, in addition to the mixtures without SCMs, which are not expected to show high durability, mixtures with 15% metakaolin, which potentially offer high durability, were also included. Similar to the mortar mixtures, these concrete mixtures were produced with a constant w/cm ratio of 0.485. Proportions for the concrete mixtures are presented in Table 5.

The naming convention for concrete mixtures follows the same interpretation method as that of mortar mixtures. For instance, C-T12-MK15 indicates a concrete mixture with Type I/II Portland cement containing 15% metakaolin as a percentage of the total cementitious materials by mass.

### 3.3. Mixing Procedures

The following sub-sections present the mixing procedures for both mortar and concrete batches.

#### 3.3.1. Mortar Mixtures

Mortar mixing was performed according to ASTM C305 [77] in a 0.019 m^3^ capacity bucket mixer with an inclined axis of rotation. The materials for each mortar batch were placed in the mixer and thoroughly mixed before being placed into molds for casting. Initially, all the required mixing water was added to the mixer bowl. After that, the cement was added to the water, and the mixer was started at a slow speed for 30 s. Then, all of the sand was gradually added over a 30-s period while maintaining the slow mixing speed. The mixer was then stopped, and the speed was changed to medium for an additional 30 s of mixing. Following this, a 90-s rest period was allowed for the mortar. During the first 15 s of this interval, any mortar adhering to the sides of the bowl was quickly scraped down into the batch. For the remainder of the interval, the mixer bowl was covered with a lid. Finally, the mortar was mixed for another 60 s at a medium speed. For each mortar mixture, three 50-mm cubes and eight bars measuring 25 × 25 × 285 mm, with gauge studs embedded in both ends, were cast.

#### 3.3.2. Concrete Mixtures

Concrete batches were mixed according to ASTM C192 [78]. The concrete mixing procedure involved using a drum mixer with an inclined rotation axis, which had a capacity of 0.06 m^3^. To initiate the mixing, the coarse aggregates and a portion of the water were added to the mixer and the mixer was started. After 30 s, the sand, cementitious materials, and remaining water were added gradually with the mixer running. The concrete batches were mixed for three minutes and then allowed to rest for three minutes with the mixer drum covered with a lid. Mixing was then continued for two additional minutes. For each concrete mixture, three cylinder specimens measuring 102 by 203 mm and six prism specimens measuring 76 × 76 × 254 mm, with gauge studs embedded at each end, were cast.

### 3.4. Sulfate Attack Testing

This study employed four different sulfate attack testing methods. For mortar mixtures, the methods included ASTM C1012 [42] and an accelerated testing approach. For concrete mixtures, an ASTM C1012-based method and an accelerated testing method were used to assess and characterize the sulfate resistance of the mixtures.

#### 3.4.1. ASTM C1012 Method

The ASTM C1012 [42] testing method was used to evaluate the sulfate resistance of mortar mixtures. For each mortar mixture, four out of the eight mortar bars and three cubes were selected for testing. Prior to demolding, specimens were initially cured according to the procedures described in ASTM C1012 [42]. During this initial curing, the specimens were placed 25 mm above a water bath. The water temperature was maintained at 35 ± 3 °C.

After 23.5 ± 0.5 h, the specimens were demolded and placed in a limewater tank at 23 ± 2 °C for a three-day curing period. At the age of four days, three mortar cubes were removed from the tank, cooled to ambient temperature under moist cloths, and tested for compressive strength according to ASTM C109 [79]. The findings of Aguayo’s [23] study showed that a four-day curing period is sufficient to initiate the sulfate tests for mortar mixtures, including those containing SCMs. Once the four-day compressive strengths were recorded, initial length measurements were conducted using a length comparator with a precision of 0.0025 mm, and the mortar bars were submerged in a container filled with a 5% sulfate solution (33,800 ppm SO_4_^2−^). To maintain a ratio of 4.0 for the volume of solution to the volume of mortar bars, each container (150 × 150 × 300 mm) contained 3000 mL of the sulfate solution.

To monitor expansion, length measurements were recorded at, or more frequently than, 1, 2, 3, 4, 8, 13, and 15 weeks. If a gradual and slight increase in expansion was observed during the specified intervals or if the specimens did not fail after 15 weeks, additional readings at 4, 6, and 9 months were scheduled to provide a comprehensive evaluation of the long-term performance of the mortar bars in the sulfate solution. It should be noted that the sulfate solution was replaced at each reading interval to provide a consistent environment of sulfate exposure and to compensate for leaching.

Following the completion of the ASTM C1012 [42] testing, the expansion results were analyzed using the criteria outlined in ACI 318-19 [80] to classify the different mortar specimens based on their sulfate resistance. Table 6 presents the maximum expansion limits specified by ACI 318-19 [80] for exposure classes S1, S2, and S3. The severity of exposure increases from class S1 to S3, with tighter expansion limits being required for mixtures exposed to more severe environments.

#### 3.4.2. ASTM C1012-Based Method

The testing method ASTM C1012 [80], originally designed for mortar mixtures, was modified to adapt it for concrete mixtures. This modified method will be referred to as the ASTM C1012-based method from this point forward. In this method, instead of using constant ratios for cement, water, and sand as specified for mortar mixtures in ASTM C1012 [42], the concrete mixtures were produced using designed mixture proportions. To accommodate coarse aggregates, the dimensions of the specimens were increased to 76 × 76 × 254 mm.

In this method, three out of the six prisms and three cylinders were selected from the specimens produced for each concrete mixture. After casting the concrete specimens, an initial curing process, similar to that of ASTM C1012 [42], was conducted on the concrete specimens. Then, the prisms were tested for compressive strength according to ASTM C39 [81]. As indicated by Aguayo’s research [23], a four-day curing period for concrete is considered adequate to initiate the sulfate tests. After measuring the four-day compressive strengths, the initial length measurements of the prisms were recorded with a length comparator that has a precision of 0.0025 mm, and the concrete prisms were immersed in a 5% sodium sulfate solution (33,800 ppm SO_4_^2−^). To maintain a ratio of 4.0 for the volume of solution to the volume of concrete prisms in the containers, each container (150 × 300 × 600 mm) was filled with 18,000 mL of sulfate solution. Finally, length measurements, conducted according to ASTM C1012 [42], were documented for all concrete prisms.

#### 3.4.3. Accelerated Testing Method

An accelerated testing method was also used to assess both mortar and concrete specimens. Four out of the eight mortar bars and three out of the six concrete prisms produced for each mortar and concrete mixture were selected for the accelerated tests. Some initial steps in the accelerated testing method were similar to the ASTM C1012 [42] procedures for mortar and the ASTM C1012-based method for concrete, such as mixing, casting, initial curing before demolding, preparation of the sulfate solution, and conducting four-day compression tests. However, after recording the four-day compressive strengths, specimens were placed in an oven at a temperature of 35 °C for 14 days. This controlled drying period was intended to remove any residual moisture from the void spaces to facilitate more complete sulfate impregnation. This step aimed to expedite the effects of sulfate exposure, enabling a more accelerated evaluation of sulfate resistance.

**Figure 1:**
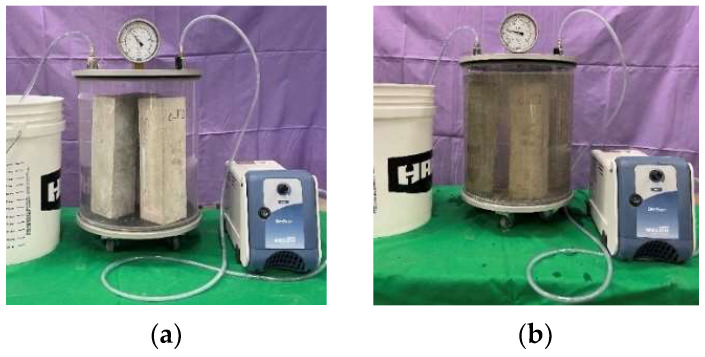
Vacuum setup for concrete prisms (**a**) before sulfate introduction and (**b**) after sulfate introduction.

At the end of the 14-day drying period in the oven, a length comparator with a precision of 0.0025 mm was used to measure the dry length of each specimen. After recording this, specimens were placed in a vacuum chamber, and the chamber was sealed (Figure 1a). Then, a vacuum pump was used to rapidly decrease the chamber internal pressure to less than 8000 Pa to expel the air within the specimens. This vacuum pressure was selected based on a previous study [23]. The vacuum pressure was maintained for four hours. After four hours of vacuum pressure, the sulfate solution was introduced by attaching a free hose on the vacuum chamber to the sulfate solution container and opening the valve (Figure 1b). The negative pressure of the vacuum chamber was used to siphon the sulfate solution into the vacuum chamber and impregnate the specimens. When the sulfate solution had fully submerged the specimens, the valve was closed. Vacuum pressure was then maintained for another 20 h to ensure thorough sulfate impregnation. This vacuum method reduces air pressure within the concrete pores, allowing sulfate solution to penetrate deeply and uniformly into the specimen. This accelerated penetration increases the rate of sulfate attack progression in the specimen. This process leads to more uniform distribution of sulfate solution throughout the specimen, more uniformly distributed expansion and cracking, and more complete exhaustion of reactants during the testing period. Finally, the vacuum was deactivated, the chamber was depressurized, and the specimens were carefully removed.

After removing the specimens from the vacuum chamber, the initial length of each specimen was measured and recorded using a digital length comparator. Specimens were then transferred to a container that contained a 5% sodium sulfate solution (33,800 ppm SO_4_^2−^). The remaining steps of the test including the requirements for the containers, solution volume, and reading intervals were conducted following the ASTM C1012 [42] for mortar and ASTM C1012-based method for concrete specimens.

## 4. Results and Discussion

This section presents the findings from sulfate attack tests conducted on mortar bars and concrete prisms. The testing methods used in this study included ASTM C1012 [42] for mortar, the ASTM C1012-based method for concrete, and an accelerated sulfate attack testing method applied to both mortar and concrete specimens.

### 4.1. Mortar Sulfate Attack Tests

#### 4.1.1. ASTM C1012 Method

Table 7 provides a summary of the results obtained for all of the mortar mixtures. The compressive strength results show that all of the mortar mixtures achieved the target strength of 20 MPa within the four-day curing period. The mortar mixture containing 30% SCM (22.5% metakaolin and 7.5% fly ash) had the greatest compressive strength. The six- and nine-month expansion results, as well as the average time required for specimens to reach the 0.05 and 0.1% expansion limits when tested according to ASTM C1012 [42], are presented because they are needed to classify a mixture into one of the sulfate exposure classes specified by ACI 318-19 [80]. For the accelerated testing method, Table 7 reports the average time required for specimens to reach the 0.1% expansion. This is because all of the specimens reached 0.1% expansion substantially earlier than the six-month mark in the accelerated testing conditions.

##### Cement Type

According to Table 7, mixtures without SCM (M-T1 and M-T2) tested according to ASTM C1012 were not suitable for any of the exposure classes specified by ACI 318-19, since they exceeded the maximum expansion limits for each of the sulfate exposure classes (0.1%) before six months of exposure to sulfate solution. These results indicate unsuitability for sulfate-rich environments. However, comparing the results from mixture M-T1 and M-T2 shows that the Type I/II mixture (M-T2) required a longer time to reach the expansion limits compared to the Type I mixture (M-T1), indicating that Type I/II cement provided greater sulfate resistance than Type I cement, as expected. The greater sulfate resistance of Type I/II cement is primarily due to its lower C_3_A content as shown in Table 3, resulting in reduced formation of expansive ettringite and consequently a decreased susceptibility to sulfate attack.

##### Silica Fume

Replacing 7.5% of Type I cement with silica fume in mixture M-T1 to produce mixture M-T1-SF7.5 did not improve sulfate resistance according to the ASTM C1012 results. Similar to the mixture M-T1, mixture M-T1-SF7.5 was not suitable for any of the exposure classes since the expansion of 0.1% was observed before the six-month mark, indicating that replacing 7.5% of Type I cement with silica fume in mixture M-T1 to produce mixture M-T1-SF7.5 was not able to increase sulfate resistance in Type I mortar mixtures. Previous research has indicated that while silica fume generally reduces expansion in mixtures, exceeding an optimal amount can lead to poorer performance [82,83].

The reason that silica fume failed to improve the sulfate resistance of this mortar mixture (M-T1-SF7.5) appears to be the inadequate dispersion of densified silica fume particles within the mortar mixture, which is an issue that has been identified by other researchers [84,85]. This lack of dispersion can be caused by the absence of coarse aggregates in mortar mixtures, which typically help to break up the densified silica fume particles. Consequently, the condensed silica fume in the mortar mixture tended to primarily act as filler rather than actively participating in chemical reactions to improve the microstructure. In certain cases (similar to mixture M-T1-SF7.5), the inclusion of silica fume may even have a negative impact on the mortar mixture. This adverse effect can be attributed to two factors: (1) the partial replacement of Portland cement with an ineffective SCM that may not have dispersed effectively or undergone complete reaction (less cement content) and (2) the presence of ultra-fine silica fume particles without pre-analyzing their effects on particle gradation can negatively affect the particle gradation in the mixture. Consequently, the presence of these ultra-fine particles can increase permeability, leading to the increased ingress and movement of sulfate ions, resulting in a detrimental effect on sulfate resistance.

##### Metakaolin

Generally, replacing cement mass with metakaolin (7.5, 15, and 22.5%) improved the sulfate resistance when using the ASTM C1012 [42] testing method. Out of the 14 mortar mixtures in this study, only 3 mixtures were suitable for all sulfate exposure classes (S1, S2, and S3) specified by ACI 318-19. Interestingly, all of these promising mixtures contained either metakaolin or a combination of metakaolin and another SCM.

As shown in Table 7, the Type I/II mixture with 15% metakaolin (M-T12-MK15) had the lowest six-month expansion (0.020%) compared to all other mortar mixtures. Although the Type I mixture with 22.5% metakaolin (M-T1-MK22.5) also had a relatively low six-month expansion (0.037%), this expansion was greater than that of M-T12-MK15. The reason for the increased expansion between these two mixtures is unclear, possibly due to the change from Type I/II cement to Type I, or the increase in metakaolin content from 15 to 22.5%. Moreover, the Type I mixture with 15% metakaolin (M-T1-MK15) showed greater six-month expansion (0.067%) compared to the Type I/II mixture (M-T12-MK15), showing that the type of cement influences the expansion. These findings align with other studies that have shown that increasing metakaolin content (as a replacement of the cement) improves sulfate resistance (reduces expansion) in sulfate-rich environments, with optimal replacement levels between 5 and 15% [64,82,86,87] and potential benefits extending up to 25% [82,88].

The improved sulfate resistance with using metakaolin can be attributed to multiple factors. Firstly, metakaolin’s high SiO_2_ and Al_2_O_3_ content makes it chemically distinct from other SCMs as it produces additional hydrates, including calcium aluminate hydrates and aluminosilicate hydrates, alongside CSH when it reacts with CH [82,89]. Secondly, its ultra-fine particles fill voids in the concrete matrix, creating a denser and less permeable microstructure, which limits sulfate ion ingress. Thirdly, metakaolin reacts with CH in the presence of water, forming additional CSH that further densifies the pore structure and limits sulfate ion movement. Additionally, the pozzolanic reaction of metakaolin with CH reduces CH availability for sulfate attack, improving sulfate resistance [90,91,92].

In the case of Type I mixtures, the addition of 7.5% metakaolin (M-T1-MK7.5) led to considerable sulfate resistance improvements. However, this mixture (M-T1-MK7.5) still did not meet the requirements of ACI 318-19 [80] for any of the exposure classes, with 0.1% expansion occurring before six months. However, increasing metakaolin content from 7.5 to 15% resulted in the specimens transitioning from being unsatisfactory for any of the exposure classes to meeting the acceptance criteria for class S1. A further increase in the metakaolin content from 15 to 22.5% (M-T1-MK22.5) ensured suitability for all sulfate exposure classes defined by ACI 318-19 [80].

Within the Type I/II mixtures, replacing 7.5% of the Type I/II Portland cement with metakaolin significantly increased sulfate resistance compared to the mixture without metakaolin (M-T12). However, it was not sufficient to render the specimens suitable for any of the sulfate exposure classes, since the 0.1% expansion occurred before six months. This finding was similar to the results obtained using Type I cement (M-T1-MK7.5). Notably, when metakaolin content was increased from 7.5 to 15%, further improvement in sulfate resistance was observed, and specimens from this mixture (M-T12-MK15) became suitable for all sulfate exposure classes specified by ACI 318-19.

**Figure 2:**
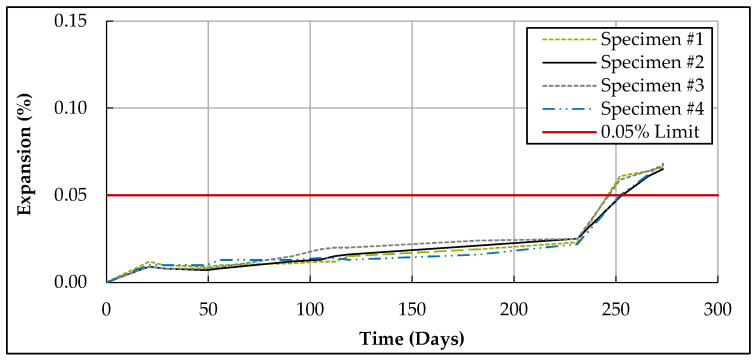
Expansion results for the mixture M-T12-MK15 using ASTM testing method.

Figure 2 illustrates the expansion versus time for mixture M-T12-MK15 when tested according to ASTM C1012 [42]. This graph reveals that the expansion increased at a moderate rate until reaching 0.025% expansion (at approximately 230 days), followed by a significant increase in slope, indicating the initiation of cracks in the specimens. This pattern of a slight plateau followed by an abrupt slope increase due to cracking was consistent among the other specimens tested according to ASTM C1012 [42,93].

##### Natural Pozzolan

Using natural pozzolan (pumicite) to replace cement mass in mortar mixtures generally improved sulfate resistance. This improvement can be attributed to the ability of natural pozzolan (similar to metakaolin) to decrease permeability and improve pore structure, and the decreased CH and C_3_A contents (per unit volume) caused by using less Portland cement [90,91,92,94,95].

Within Type I mixtures, although increasing the natural pozzolan content from 0 to 7.5% or from 7.5 to 15% improved sulfate resistance, replacing either 7.5 or 15% of cement with natural pozzolan did not lead to a change in exposure classes. Both M-T1-NP7.5 and M-T1-NP15 mixtures reached the 0.1% expansion limit before the six-month mark, indicating unsuitability for any sulfate exposure classes, similar to the mixture without natural pozzolan (M-T1). These findings are consistent with the results from M-T1-MK7.5, where 7.5% of the cement mass was replaced with metakaolin. This shows that replacing 7.5% of the Type I cement mass with SCMs, whether it is natural pozzolan or metakaolin, is insufficient to achieve sulfate resistance levels that meet the requirements for any of the sulfate exposure classes. However, metakaolin showed a superior effect in improving sulfate resistance compared to natural pozzolan.

In the case of Type I/II mixtures, replacing 15% of Type I/II cement with natural pozzolan (mixture M-T12-NP15) significantly improved sulfate resistance, making it suitable for sulfate exposure class S1 according to ACI 318-19. When the ASTM C1012 [42] method was used, M-T12-NP15 specimens reached the 0.05 and 0.1% expansion limits after 157 and 250 days, respectively. Since the expansion after 180 days was less than 0.1% (0.055%), this mixture could be considered suitable for sulfate exposure class S1 as specified by ACI 318-19 [80]. However, to accept this mixture for exposure classes S2 and S3, the expansion at 12 months would need to be less than 0.1%. Nine-month testing showed that specimens from this mixture reached 0.1% expansion prior to 12 months (at 250 days), indicating that this mixture cannot be accepted for sulfate exposure classes S2 and S3 as defined by ACI 318-19 [80].

Comparing Type I and Type I/II mortar mixtures with 15% natural pozzolan (M-T1-NP15 and M-T12-NP15) indicates that the use of Type I/II Portland cement significantly increased the testing time and transitioned the specimens from being unsuitable for any of the sulfate exposure classes (Type I mixture) to meeting the requirements for exposure class S1 (Type I/II mixture). This is primarily attributed to the lower C_3_A content in the Type I/II cement compared to the Type I cement (Table 3), resulting in a reduced formation of expansive ettringite.

##### SCMs Combination

The results showed that the specimens that contained a combination of 7.5% metakaolin and 7.5% silica fume (M-T1-MK7.5SF7.5 and M-T12-MK7.5SF7.5), tested according to ASTM C1012 [42], did not meet the requirements outlined by ACI 318-19 [80] for any of the sulfate exposure classes since the expansion of 0.1% occurred in less than six months.

Another mortar mixture, M-T1-MK22.5FA7.5, containing a combination of SCMs (22.5% metakaolin and 7.5% fly ash) showed expansions of 0.023 and 0.054% after 180 and 270 days of testing, respectively, when subjected to the ASTM C1012 [42] method. Since the expansion was less than 0.1% after six months (0.023%), the mixture M-T1-MK22.5FA7.5 met the requirements for exposure class S1 according to ACI 318-19 [80]. Additionally, since the six-month expansion was less than 0.05%, the specimens were suitable for exposure classes S2 and S3 (more severe sulfate exposure classes than S1). These findings indicate that the mixture M-T1-MK22.5FA7.5 outperformed other mortar mixtures in terms of sulfate resistance (least expansion) and was one of the only two Type 1 mortar mixtures, along with M-T1-MK22.5, that met the requirements for all exposure classes. Other studies have also shown that fly ash and metakaolin significantly enhance sulfate resistance in sodium sulfate environments [82,83,96]. For Class F ashes, a decreasing sulfate attack expansion has been observed with an increasing fly ash replacement, up to 80% [83,96]. This exceptional performance can be attributed to various factors, including the incorporation of fly ash, the synergistic effects of the two SCMs (metakaolin and fly ash), and the overall greater content of SCMs in the mixture (30%) in decreasing permeability, improving pore structure, and reducing CH and C_3_A contents (per unit volume).

Comparing mixture M-T1-MK22.5FA7.5 and mixture M-T1-Mk22.5 indicates that the additional 7.5% fly ash in a mixture already containing 22.5% metakaolin significantly prolonged the testing time (improved sulfate resistance). The specimens with fly ash (M-T1-MK22.5FA7.5) showed approximately half the expansion observed in mixtures without fly ash (M-T1-Mk22.5) at the same testing age (0.037% compared to 0.023% for a 180-day testing period and approximately 0.1% compared to 0.054% for a 270-day testing period).

#### 4.1.2. Accelerated Method (Mortar Mixtures)

Table 7 reveals the significant time-saving benefits of the accelerated testing method for assessing sulfate resistance compared to the ASTM C1012 [42] method. Notably, it effectively reduced the duration needed to reach the 0.05 and 0.1% expansion limits (testing time) for various mixtures. For instance, it reduced the time required to reach the 0.1% expansion limit from 21.3 to 3.67 days for mixture M-T1 and from 105 to 17.8 days for mixture M-T12-MK7.5 when compared with the ASTM C1012 [42] tests. In addition to the time-saving advantages of the accelerated procedure, the results from the accelerated tests for mortar mixtures showed a strong correlation with the results from the ASTM C1012 tests. For example, the use of Type I/II cement, rather than Type I cement, consistently increased the testing time in both the accelerated and ASTM C1012 tests. Similarly, when cement was replaced with metakaolin, the accelerated procedure showed an increased testing time, consistent with the results observed within the ASTM C1012 tests.

##### Characterizing Mortar Sulfate Resistance Based on the Accelerated Method

The accelerated testing method employed in this study is not a standardized test. Therefore, it is necessary to develop a method for interpreting the results obtained from this accelerated test to characterize the sulfate resistance of mortar mixtures. The proposed approach was selected to ensure a reasonable correlation between the results obtained from ASTM C1012 [42] and the accelerated testing method. The proposed interpretation for the accelerated test results is based on expansions after 14 and 21 days of sulfate exposure following vacuum impregnation.

Table 8 presents the criteria used to interpret the accelerated test results. These criteria were intended to provide an effective characterization process for the limited dataset generated during this research that correlates with the classification system applied by ACI 318-19 [80] to results from ASTM C1012 [42]. These criteria are also proposed as a starting point for any future research that builds on this dataset.

Table 9 presents the 14- and 21-day expansion results for mortar mixtures subjected to the accelerated testing method. Table 9 also includes a description of the sulfate resistance of the mortar mixtures, using both the ACI 318-19 [80] interpretation of the ASTM C1012 [42] results and the proposed method for interpretation of the accelerated test results.

One way to evaluate the consistency between the accelerated testing method and the standard ASTM C1012 [42] is by comparing the sulfate resistance classifications obtained from both approaches. As shown in Table 9, the proposed method for interpreting the accelerated test results was consistent with the classification method presented by ACI 318-19 [80] for mixtures with low sulfate resistance according to the accelerated testing. Specifically, the Type I and Type I/II mixtures without SCM, the Type I mixture with 7.5% silica fume, and Type I mixtures with 7.5 and 15% natural pozzolan showed a low sulfate resistance based on the accelerated method. These mixtures were similarly unsuitable for sulfate exposure according to ACI 318-19 [80] as determined from the results from ASTM C1012 [42] testing.

Although the accelerated and ASTM C1012 [42] results for mixtures with moderate and high sulfate resistance were acceptably consistent, the consistency was not as good as for the mixtures with low sulfate resistance, and some discrepancies were observed for specific mixtures. However, none of the deviations had differences that were greater than one level of sulfate resistance. For instance, the Type I and Type I/II mixtures with 7.5% metakaolin or with a combination of metakaolin and silica fume were classified as unsuitable for any exposure class based on the ASTM method, but showed moderate sulfate resistance according to the accelerated method, representing a deviation (differing by one level) from the ASTM C1012 [42] results. As another example, the Type I mixture with 15% metakaolin had a high sulfate resistance based on the accelerated test but was considered suitable for only sulfate exposure class S1 according to the ACI 318-19 [80] interpretation of ASTM C1012 [42], presenting the lone discrepancy between the interpretations of the two methods for mixtures in the high sulfate resistance category.

Perhaps a better way to assess the consistency between the accelerated testing method and standard ASTM C1012 [42] in mortar mixtures is by analyzing the time required to reach 0.1% expansion in the ASTM C1012 test and comparing it to the sulfate resistance classifications from the accelerated method. The results of this comparison are presented in Table 10 and reveal a strong correlation between the results of the two methods. Specifically, there is no overlap in the age range for 0.1% expansion when mixtures were tested according to ASTM C1012 [42] across the three sulfate resistance classifications (low, moderate, and high) from the accelerated testing. This comparison provides an interpretation where results from the two test methods can be categorized similarly with 100% reliability for the small dataset produced in this work. This type of analysis may prove useful in future work for establishing revised classification criteria for the accelerated test; however, expansion results were used to establish classification criteria in this work to be consistent with ACI 318-19 [80] criteria. A similar comparison should not be made quantitively using expansion measurements from the two tests, because the two tests are not equally accurate or effective. Since the accelerated test produces expansion that is more uniformly distributed throughout the specimens and the sulfate reaction nears exhaustion, the accelerated test can be justified as being the better test for assessing the sulfate resistance of mortars.

In conclusion, the proposed approach for interpreting the accelerated test results offers a promising direction for efficiently classifying sulfate resistance within a relatively short timeframe (around a month). However, this approach requires further research and validation to establish its effectiveness. Conducting a round-robin study, involving multiple labs and multiple users running the test, would be appropriate to validate the results and assess the consistency of the data across different settings. Additionally, by considering multiple factors, such as compressive strength, permeability, and vacuum pressure, and adopting a comprehensive microstructural evaluation, a more reliable assessment of sulfate resistance in mortar mixtures containing SCMs might be achieved.

### 4.2. Concrete Sulfate Attack Tests

In this study, two methods for assessing concrete sulfate performance were proposed: one based on ASTM C1012 [42], and an accelerated testing method. Since these methods are not standardized, it is important to develop effective approaches for interpreting the results derived from these testing methods.

Table 11 presents a summary of the results obtained for all four concrete mixtures in this study. The results indicate that the compressive strength of all concrete mixtures was at least 38.0 MPa prior to initiating length measurements. This compressive strength is greater than that of the mortar mixtures reported in Table 7; however, compressive strength was not the criterion for initiating these tests; only the special curing for four days (described in Section 3.4) was required. The average time taken by concrete specimens to reach the 0.04% expansion was also provided in Table 11 because it is a well-stablished expansion limit for ASR testing according to ASTM C1778 [97]. Additionally, the age at which the expansion reached 0.1%, as well as the expansions at six and nine months, were recorded for the concrete specimens tested using the ASTM C1012-based method. For the accelerated testing method, Table 11 provides the average times required for concrete specimens to reach the 0.04 and 0.1% expansion limits. This allows for a comparison of the testing duration for a specific expansion threshold between the ASTM C1012-based method and the accelerated testing method.

#### 4.2.1. ASTM C1012 Based Method

As indicated in Table 11, the concrete mixtures without SCMs (C-T1 and C-T12) exceeded the 0.04% expansion threshold before the six-month mark, indicating potential unsuitability for sulfate-rich environments. As expected, the use of Type I/II cement improved sulfate resistance compared to Type I cement due to the lower C_3_A content in Type I/II cement. It is worth mentioning that mortar bars with similar cementitious material combinations (M-T1 and M-T2) similarly failed to meet the acceptance criteria for any of the sulfate exposure classes specified in ACI 318-19 [80].

Replacing 15% of cement with metakaolin (C-T1-MK15 and C-T12-MK15) significantly increased the time required to reach the expansion thresholds (improved sulfate resistance). Mixtures C-T1-MK15 and C-T12-MK15 showed expansions of 0.027 and 0.010% after six months of sulfate exposure, respectively. The observed improvement in sulfate resistance with the inclusion of 15% metakaolin can be attributed to its ability to reduce permeability, enhance pore structure, and reduce CH and C_3_A contents (per unit volume) by using less Portland cement [90,91,92,94,95].

For mixture C-T1-MK15, the six-month expansion was significantly below the 0.04% expansion limit, but the nine-month expansion was relatively high (0.067%). This suggests that this mixture may be suitable for relatively mild sulfate exposure but might not be ideal for high sulfate concentrations. Comparing this mixture with the mortar mixture that had the same combination of cementitious materials (M-T1-MK15) indicated that both mixtures reached approximately 70% of their respective expansion limits (0.1% for mortar and 0.04% for concrete) at six months. The same trend was observed when comparing C-T12-MK15 and M-T12-MK15, with both reaching around 25% of their respective expansion limits. This consistency provides support for the ability to apply the ASTM C1012-based testing method for assessing the sulfate resistance of concrete mixtures.

Mixture C-T12-MK15 showed an exceptionally low six-month expansion (less than half of the 0.04% expansion limit). This indicates excellent sulfate resistance, making it well suited for exposure to high sulfate concentrations. This conclusion is drawn from a proportional interpretation of ACI 318-19 [80], where mortar mixtures with six-month expansion less than half of the 0.1% expansion limit qualify for all sulfate exposure classes.

The finding that mixture C-T12-MK15 outperformed the concrete mixture C-T1-MK15 was consistent with the results of comparing mortar mixtures M-T1-MK15 and M-T12-MK15. This consistency emphasizes the beneficial effect of using Type I/II cement in combination with metakaolin to improve the sulfate resistance properties of cementitious materials in both concrete and mortar mixtures.

##### Characterizing Concrete Sulfate Resistance Using the ASTM-Based Method

Table 12 presents the proposed classification criteria selected for the ASTM C1012-based method for concrete mixtures. These criteria were selected using the concepts that ACI 318-19 [80] uses to interpret ASTM C1012 [42] test results for mortar mixtures. The primary modification from the ACI 318-19 [80] criteria is that the expansion limits were revised from 0.05 and 0.1% in ACI 318-19 [80] to 0.02 and 0.04% to interpret results from the ASTM C1012-based method. In this proposed interpretation method, the sulfate resistance of the concrete mixtures was evaluated by observing the six-month expansion, with 0.02 and 0.04% expansions serving as the threshold values. As stated earlier, the 0.04% expansion was selected because it is a well-stablished expansion limit for concrete ASR testing according to ASTM C1778 [97].

Table 13 provides the six-month expansions that occurred for concrete mixtures using the ASTM C1012-based method. Additionally, Table 13 presents the sulfate resistance classification for the concrete mixtures by applying the proposed interpretation method (Table 12) to the results from the ASTM C1012-based tests.

Comparing the results in Table 11 and Table 13 shows that the proposed interpretation method in Table 12 for concrete mixtures yields results consistent with the ACI 318-19 [80] interpretation for mortar mixtures with similar cementitious material combinations. Specifically, both mortar and concrete mixtures without SCM were unsuitable for sulfate exposure. Additionally, the Type I mortar mixture containing 15% metakaolin (M-T1-MK15) was classified as suitable for sulfate exposure class S1 as shown in Table 11. The comparable concrete mixture (C-T1-MK15) was similarly considered suitable for moderate sulfate exposure as stated in Table 13. The Type I/II mortar mixture with 15% metakaolin (M-T12-MK15) was also found to be appropriate for all sulfate exposures as indicated in Table 11. Likewise, the comparable concrete mixture (C-T12-MK15) was considered acceptable for use in environments with high sulfate concentrations, as stated in Table 13.

#### 4.2.2. Accelerated Method (Concrete Mixtures)

Table 11 shows the substantial time-saving advantages of the accelerated testing method compared to the ASTM-based method for concrete. It effectively reduced the duration required to reach the 0.04 and 0.1% expansion limits (testing time) for various mixtures. For instance, it decreased the time needed to reach the 0.1% expansion limit for mixtures C-T1 and C-T2 (mixtures without SCM) from 181 to 72.9 days and from 263 to 100 days, respectively, compared to the ASTM-based tests. This resulted in an average reduction of approximately 60% in the time required to achieve a specific expansion threshold when the accelerated testing method was employed, as opposed to the ASTM-based method. 

In addition to the time-saving benefits of the accelerated procedure, the results from the accelerated concrete tests showed a strong correlation with the results from the ASTM C1012-based tests. Specifically, concrete mixture C-T12 consistently required more time to reach expansion limits (indicating greater resistance to sulfate attack) compared to mixture C-T1, as expected due to the lower C_3_A content in Type I/II cement. Similarly, the use of 15% metakaolin in the concrete mixture to replace cement mass increased the accelerated testing time compared to mixtures without SCM, consistent with the results from the ASTM C1012-based tests.

##### Characterizing Concrete Sulfate Resistance Using the Accelerated Method

Table 14 presents the proposed criteria to interpret the results from the accelerated concrete tests. These criteria were selected to categorize concrete sulfate resistance using the accelerated testing approach in a manner that provides a reasonable correlation with the ASTM C1012-based test results. The evaluation of sulfate resistance in this method was based on the accelerated concrete expansions after 28 and 56 days of exposure to sulfate solution.

Table 15 provides the 28- and 56-day expansions for concrete mixtures tested using the accelerated testing method. Additionally, Table 15 provides the sulfate resistance classification for these concrete mixtures by applying the proposed interpretation (Table 14) to the accelerated test results.

The sulfate resistance descriptions for concrete mixtures tested using the accelerated testing method (Table 15) were in complete agreement with the descriptions from the ASTM C1012-based tests (Table 13). For instance, concrete mixtures without SCM (C-T1 and C-T12) were found to be unsuitable for sulfate exposure due to their 28-day accelerated expansions exceeding 0.04%, which is consistent with the observations from the ASTM C1012-based tests (Table 13). As another example, the Type I/II concrete mixture containing 15% metakaolin (C-T12-MK15) was shown to be suitable for exposure to high sulfate concentrations in both the accelerated and ASTM C1012-based tests.

To further assess the consistency between the accelerated testing method and ASTM C1012-based method in concrete mixtures, the time to reach 0.04% expansion in the ASTM C1012-based method can be compared with sulfate resistance classifications from the accelerated method. According to Table 16, which presents this comparison, the time needed to reach 0.04% expansion in the ASTM C1012-based tests can be clearly delineated between the three sulfate resistance classifications in the accelerated test in a manner that appears to be 100% reliable for the small dataset produced for this work.

The high correlation between the accelerated testing method (Table 15) and the ASTM C1012-based approach (Table 13) highlights the repeatability and reliability of both proposed classification criteria (Table 12 and Table 14) for evaluating sulfate resistance in concrete mixtures using the ASTM C1012-based tests and accelerated tests. However, it is crucial to emphasize that these approaches to interpreting the results from accelerated tests and ASTM C1012-based tests require further research and validation to establish their effectiveness and reliability. To ensure the robustness of the results and the consistency of the data across various settings, a round-robin study involving multiple laboratories conducting the test would be appropriate.

### 4.3. Correlation between ASTM and Accelerated Testing Methods

One method to evaluate the repeatability and reliability of the testing methods applied in this study is by analyzing the standard deviation (SD) of the results. The SD is influenced by multiple factors, including the average value of the samples, individual sample values, and the number of samples. Given that ASTM C1012 typically involves longer test durations and higher average values compared to the accelerated testing method, its SD values are generally greater. However, these greater SD values do not necessarily indicate greater variability in the ASTM C1012 results compared to the accelerated tests. The increased SD in ASTM C1012 is more a consequence of its longer test duration (greater values for samples and means) rather than actual variability.

**Figure 3:**
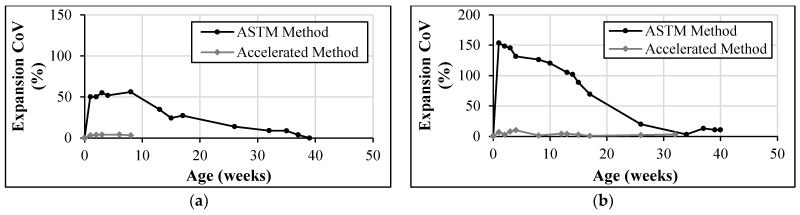
Expansion CoV for the mixtures (**a**) M-T12-NP15, and (**b**) C-T1-MK15.

To avoid the bias associated with SD, the coefficient of variation (CoV), which normalizes the SD relative to the mean and provides a more consistent measure of relative variability, can be used. This approach ensures a fair comparison between the ASTM C1012 and accelerated testing methods by using normalized data, improving the clarity and accuracy of the analysis. In this study, the coefficient of variation in expansion (expansion CoV) was evaluated at different testing stages. Figure 3 provides examples of this analysis, visually presenting the expansion CoV values at each testing interval during the experiments.

The findings show that the expansion CoV values obtained from the accelerated testing method were generally lower or comparable to those from the ASTM C1012 [42] method for mortar mixtures and the ASTM C1012-based method for concrete mixtures. This signifies a higher level of repeatability in the accelerated tests compared to the ASTM C1012 [42] method for mortar mixtures and the ASTM C1012-based method for concrete mixtures.

**Figure 4:**
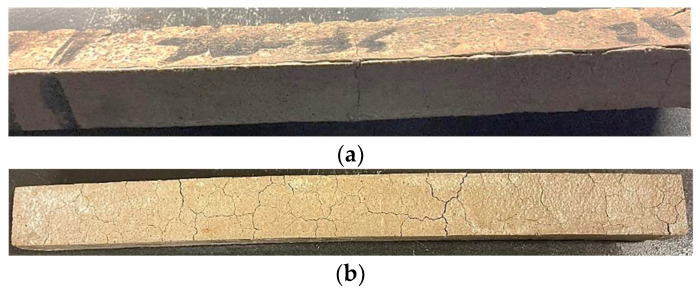
Dominant crack formation in mortar specimens using (**a**) ASTM and (**b**) accelerated testing methods.

This improved repeatability for the accelerated tests, indicated by low CoV values, can be attributed to the vacuum impregnation providing more uniform distribution of sulfate solution throughout each specimen, causing more distributed progression of the sulfate reaction and more consistent expansions. Distinctly different modes of deterioration were associated with each method. In the ASTM C1012 [42] testing for mortar and the ASTM C1012-based testing for concrete mixtures, the presence of large surface cracks, known as diffusion-controlled cracking (Figure 4a), facilitated the ingress of sulfate solution, resulting in rapid localized expansion. In contrast, the accelerated method employed vacuum impregnation to induce sulfate ingress, leading to more uniform expansion throughout the specimen. This, in turn, resulted in distributed internal cracking, subsequent map cracking on the surface (Figure 4b), and more complete exhaustion of reactants. The lower CoV values, caused by the uniform reaction throughout the specimen, not only Indicate an improved repeatability for the accelerated test, but also that the accelerated tests are more consistent and reliable than the non-accelerated tests.

## 5. Conclusions

This study compares four testing methods for assessing sulfate resistance in mortar and concrete mixtures, including accelerated and standard ASTM C1012 methods for mortar, as well as accelerated and ASTM C1012-based methods for concrete, with the purpose of further developing the accelerated tests. A total of 14 mortar mixtures and 4 concrete mixtures using different SCMs were produced to provide a reasonably broad selection of mixtures that would facilitate evaluation of the effectiveness of the newly developed tests. In the accelerated testing methods, vacuum pressure was used to produce deep penetration of the sulfate solution into the specimens, leading to rapid and nearly complete exhaustion of the reactants. This process causes uniform expansion throughout the specimens and small distributed cracks, as opposed to the large, localized cracks observed in the non-accelerated tests.

The accelerated testing methods significantly reduced the testing time compared to the non-accelerated testing methods. The accelerated methods reduced the duration from 12 months to 21 days for mortar mixtures and from six months to 56 days for concrete mixtures, reducing the testing time by as much as 70%. These methods offer promising alternatives to the standard ASTM C1012 test for evaluating sulfate resistance, which is not only time-consuming but is also limited in its application to standard mortars.

The results showed that the accelerated test for mortar mixtures, based on 14- and 21-day expansions, can be interpreted in a manner that aligns reasonably well with the sulfate resistance classifications of ACI 318-19. The proposed interpretation methods for sulfate resistance of concrete mixtures also showed excellent consistency with ACI 318-19 when compared to results from mortar mixtures using the same cementitious material combinations. Additionally, the accelerated methods generally showed lower expansion CoV values at various testing intervals for both mortar and concrete, indicating greater consistency and reliability for the accelerated testing methods compared to the non-accelerated methods. Overall, the accelerated testing methods result in more uniform expansion throughout the specimens, nearly complete exhaustion of the reactants, and lower CoV values, which combine to indicate that the accelerated testing methods provide more repeatable and reliable results than the non-accelerated testing methods.

The results from all four testing methods consistently indicated that mixtures without SCMs, whether using Type I or Type I/II Portland cement, were unsuitable for sulfate exposure. However, Type I/II cement showed greater sulfate resistance than Type I cement in both mortar and concrete mixtures, regardless of the type and concentration of SCMs used. This improvement is attributed to the lower C_3_A content of Type I/II cement. Among the SCMs tested, replacing cement with metakaolin (at 7.5, 15, or 22.5%) or natural pozzolan (at 7.5 or 15%) reasonably improved the sulfate resistance in mortar mixtures. Also, a Type I/II concrete mixture containing 15% metakaolin was particularly effective in environments with high sulfate concentrations. This improvement appears to be primarily due to the general ability of SCMs to decrease permeability and improve pore structure, as well as the reduced CH and C_3_A contents caused by using less Portland cement, even though metakaolin has been shown to produce additional calcium aluminate hydrates. However, using 7.5% silica fume in mortar mixtures did not yield similar benefits, most likely due to the inadequate dispersion of the condensed silica fume particles caused by the absence of coarse aggregates in the mortar mixtures and the consequent limited reactivity of the undispersed particles.

While this study shows the effectiveness of the accelerated testing methods, there are limitations that should be acknowledged. For instance, the dataset used to develop the interpretation criteria for the newly developed tests contained a small population of mixtures. Additionally, the provided interpretation methods in this study should be considered as preliminary, since they only classify sulfate resistance categories, not the actual exposures encountered in real-world situations. In contrast, the standard ASTM C1012 provides more practical engineering categories based on water-soluble sulfate in soil or dissolved sulfate in water, specified in ACI 318-19. Moreover, only sodium sulfate solution was used in this work, whereas other types of sulfates, which can also degrade concrete, were not explored.

## 6. Recommendations for Future Work

This research provides preliminary results for the methods developed in this study. However, further research is needed to expand the population of mixtures in a manner that incorporates a broader range of mix design parameters (different types of cement, alternative SCMs, varying SCM contents, admixture dosages, and different w/cm ratios) for both mortar and concrete mixtures. For example, future studies could investigate different sulfate solutions, such as magnesium sulfate, and use other types of cement, such as Type 1L or Type V. Additionally, investigating other types of SCMs, such as slag, and using a wider range of SCM contents in the mixtures would facilitate the revision of the interpretation criteria for the accelerated tests so that they are appropriate for a broader range of mixtures.

Since sulfate resistance can be influenced by various factors such as compressive strength, permeability, and vacuum pressure, it would also be useful to include mixtures with different compressive strengths and permeabilities in future research. Exploring different vacuum pressures to determine the sensitivity of the test results to variations in vacuum pressure is also important for further development of the accelerated test methods. Conducting microstructural evaluations, such as analyzing the chemical composition of reaction products, should also offer a better understanding of how deep the sulfate solution penetrates into the specimens and how complete the sulfate reactions are at the end of testing. Each of these future topics would improve the understanding of important details associated with the accelerated testing methods and provide opportunities to make these tests more reliable.

Repeating tests with a single operator and conducting a round-robin study involving multiple laboratories and users would help establish precision criteria for the newly developed testing methods. Assessing the consistency of results across different users or laboratories would improve the ability to assess the reliability of the testing methods and their interpretation criteria and might also identify procedural changes that could improve that reliability.

## Figures and Tables

**Table 1:** Physical properties of aggregates.

Aggregate Type	Bulk Specific Gravity	Moisture Content (%)	Absorption (%)	Dry Rodded Unit Weight (kg/m^3^)	Fineness Modulus
Coarse Agg.	2.51	0.22	0.50	1466	-
Sand	2.54	0.41	1.17	-	2.78

**Table 2:** Aggregate particle size distribution (percent passing).

Aggregate Type	Sieve Opening (mm)										
	25.4	19.0	12.7	9.5	4.75	2.36	1.18	0.6	0.3	0.15	PAN
Coarse Agg.	99.1	75.9	43.4	30.7	6.22	3.31	-	-	-	-	0.0
Sand	-	-	-	-	95.2	82.7	71.2	53.3	18.3	1.53	0.0

**Table 3:** Chemical and physical properties of cementitious materials (% mass).

Chemical Properties	Material					
	Cement Type I	Cement Type I/II	Silica Fume	Metakaolin	Pumicite	Class F Fly Ash
CaO	46.9	65.1	0.3	0.87	0.40	8.99
SiO_2_	10.47	20.5	96.9	63.86	76.29	53.16
Al_2_O_3_	2.86	4.0	0.2	31.11	12.13	24.64
Fe_2_O_3_	0.95	2.7	0.2	1.06	1.74	4.22
MgO	1.2	2.5	0.2	0.18	0.07	1.25
Na_2_O	0.78	0.3	0.2	1.08	4.23	1.66
K_2_O	-	0.4	0.3	0.09	4.29	1.24
TiO_2_	-	-	-	-	0.10	-
MnO_2_	-	-	-	-	0.08	-
P_2_O_5_	-	-	-	-	0.02	-
SrO	-	-	-	-	0.01	-
BaO	-	-	-	-	0.01	-
SO_3_	3.2	2.9	0.1	0.05	0.00	0.25
Loss on Ignition	2.2	2.6	2.17	1.18	-	-
**Chemical Composition**						
C_3_S	57	64	-	-	-	-
C_2_S	16	10	-	-	-	-
C_3_A	11	6	-	-	-	-
C_4_AF	7	8	-	-	-	-
C_3_S + 4.75 × C_3_A	107	-	-	-	-	-
**Physical Properties**						
Specific Gravity	3.15	3.15	2.20	2.60	2.45	1.91
Spec. Surface Area (m^2^/kg)	373	420	26,810	22,320	17,348	734
Autoclave Expansion (%)	0.02	0.03	-	-	-	0.01

**Table 4:** Mortar mixture proportions.

SCM Content	Mixture Name	Type I Cement (g)	Type I/II Cement (g)	SF (g)	MK (g)	NP (g)	FA (g)
No SCM	M-T1	1515	-	-	-	-	-
	M-T12	-	1515	-	-	-	-
7.5% SF	M-T1-SF7.5	1401	-	114.2	-	-	-
7.5% MK	M-T1-MK7.5	1401	-	-	114.2	-	-
	M-T12-MK7.5	-	1401	-	114.2	-	-
15% MK	M-T1-MK15	1287	-	-	228.4	-	-
	M-T12-MK15	-	1287	-	228.4	-	-
22.5% MK	M-T1-MK22.5	1174	-	-	341.0	-	-
7.5% NP	M-T1-NP7.5	1401	-	-	-	114.2	-
15% NP	M-T1-NP15	1287	-	-	-	228.4	-
	M-T12-NP15	-	1287	-	-	228.4	-
7.5% MK + 7.5% SF	M-T1-MK7.5SF7.5	1287	-	114.2	114.2	-	-
	M-T12-MK7.5SF7.5	-	1287	114.2	114.2	-	-
22.5%MK + 7.5%FA	M-T1-MK22.5FA7.5	1060	-	-	341.0	-	114.2

Note: Fine aggregate of 4166 g and water of 734 g were used for all mixtures.

**Table 5:** Concrete mixture proportions.

SCM Content	Mixture Name	Type I Cement (kg/m^3^)	Type I/II Cement (kg/m^3^)	MK (kg/m^3^)
No SCM	C-T1	416	-	-
	C-T12	-	416	-
15% MK	C-T1-MK15	354	-	62
	C-T12-MK15	-	354	62

Note: Coarse aggregate of 973 kg/m^3^, fine aggregate of 676 kg/m^3^, and water of 202 kg/m^3^ were used for all mixtures.

**Table 6:** Requirements of ACI 318-19 [80] for different sulfate exposures.

Exposure		Maximum Expansion If Tested Using ASTM C1012 [42]		
		6 Months	12 Months	18 Months
S1		0.1%	No requirement	No requirement
S2		0.05%	0.1%	No requirement
S3	Option 1	No requirement	No requirement	0.1%
	Option 2	0.05%	0.1%	No requirement

**Table 7:** Summary of mortar mixture results.

Mixture Name	Compressive Strength (MPa)	ASTM Testing Method				Accelerated Testing Method
		Age at 0.05% Expansion (Day)	Age at 0.1% Expansion (Day)	Expansion after Six Months (%)	Expansion after Nine Months (%)	Age at 0.1% Expansion (Day)
M-T1	20.3	15.4	21.3	-	-	3.67
M-T12	24.5	22.1	26.8	-	-	5.83
M-T1-SF7.5	26.2	10.3	16.8	-	-	4.01
M-T1-MK7.5	27.8	63.7	79.9	-	-	10.6
M-T12-MK7.5	26.3	80.0	105	-	-	17.8
M-T1-MK15	28.2	112	264	0.067	-	5.60
M-T12-MK15	27.4	249	>270	0.020	0.067	9.88
M-T1-MK22.5	24.9	226	263	0.037	-	5.06
M-T1-NP7.5	24.5	35.6	45.7	-	-	2.40
M-T1-NP15	25.9	49.3	63.7	-	-	1.56
M-T12-NP15	26.5	158	250	0.055	-	2.51
M-T1-MK7.5SF7.5	20.1	66.9	94.2	-	-	6.78
M-T12-MK7.5SF7.5	21.4	83.4	123	-	-	9.01
M-T1-MK22.5FA7.5	28.5	253	>270	0.023	0.054	24.9

Note: “-” indicates that the mixture reached the 0.1% expansion before six months, or the sulfate resistance of the mixture had already been categorized.

**Table 8:** Classification criteria based on the accelerated results for mortar mixtures.

Sulfate Resistance		14-Day Accelerated Expansion (%)	21-Day Accelerated Expansion (%)
High	Option 1	Expansion ≤ 0.08	No requirement
	Option 2	0.08 < Expansion ≤ 0.20	Expansion < 120% of the 14-day value
Moderate		Does not meet the requirements for either high or low resistance classes	
Low		Expansion ≥ 0.30	No requirement

**Table 9:** Sulfate resistance classification for mortar mixtures using ASTM and accelerated method.

Mixture Name	ASTM C1012 [42] Testing Method	Accelerated Testing Method		
	ACI 318-19 [80] Exposure Class	Expansion after 14 Days (%)	Expansion after 21 Days (%)	Sulfate Resistance
M-T1	-	0.696	1.201	Low
M-T12	-	0.446	0.771	Low
M-T1-SF7.5	-	0.770	1.394	Low
M-T1-MK7.5	-	0.118	0.155	Moderate
M-T12-MK7.5	-	0.087	0.111	Moderate
M-T1-MK15	S1	0.137	0.156	High
M-T12-MK15	S1, S2, and S3	0.153	0.163	High
M-T1-MK22.5	S1, S2, and S3	0.207	0.232	Moderate
M-T1-NP7.5	-	0.989	0.996	Low
M-T1-NP15	-	0.462	0.468	Low
M-T12-NP15	S1	0.288	0.292	Moderate
M-T1-MK7.5SF7.5	-	0.150	0.265	Moderate
M-T12-MK7.5SF7.5	-	0.144	0.193	Moderate
M-T1-MK22.5FA7.5	S1, S2, and S3	0.034	0.064	High

**Table 10:** Comparison between the mortar results from ASTM and accelerated methods.

Mixture Name	ASTM C1012 [42] Testing Method	Accelerated Testing Method
	Age at 0.1% Expansion (Day)	Sulfate Resistance
M-T1-SF7.5	16.8	Low
M-T1	21.3	
M-T12	26.8	
M-T1-NP7.5	45.7	
M-T1-NP15	63.7	
M-T1-MK7.5	79.9	Moderate
M-T1-MK7.5SF7.5	94.2	
M-T12-MK7.5	105	
M-T12-MK7.5SF7.5	123	
M-T12-NP15	250	
M-T1-MK22.5	263	
M-T1-MK15	264	High
M-T12-MK15	>270	
M-T1-MK22.5FA7.5	>270	

**Table 11:** Summary of concrete mixture results.

Mixture Name	Compressive Strength (MPa)	ASTM-Based Testing Method				Accelerated Testing Method	
		Age at 0.04% Expansion (Day)	Age at 0.1% Expansion (Day)	Expansion after Six Months (%)	Expansion after Nine Months (%)	Age at 0.04% Expansion (Day)	Age at 0.1% Expansion (Day)
C-T1	45.8	103	181	0.099	-	18.5	72.9
C-T12	40.8	131	263	0.058	-	27.0	100
C-T1-MK15	43.5	240	-	0.027	0.067	70.0	173
C-T12-MK15	38.0	277	-	0.010	0.042	116	209

**Table 12:** Classification criteria using the ASTM-based test results for concrete mixtures.

Sulfate Resistance	Six-Month Expansion (%)
Suitable for exposure to high sulfate concentrations	Expansion ≤ 0.02
Suitable for moderate sulfate exposure	0.02 < Expansion ≤ 0.04
Unsuitable for sulfate exposure	Expansion > 0.04

**Table 13:** Sulfate resistance classification for concrete mixtures using ASTM based method.

Mixture Name	Six-Month Expansion (%)	Sulfate Resistance
C-T1	0.099	Unsuitable for sulfate exposure
C-T12	0.058	Unsuitable for sulfate exposure
C-T1-MK15	0.027	Suitable for moderate sulfate exposure
C-T12-MK15	0.010	Suitable for exposure to high sulfate concentrations

**Table 14:** Classification criteria using the accelerated results for concrete mixtures.

Sulfate Resistance	28-Day Accelerated Expansion (%)	56-Day Accelerated Expansion (%)
Suitable for exposure to high sulfate concentrations	Expansion ≤ 0.02	No requirement
Suitable for moderate sulfate exposure	0.02 < Expansion ≤ 0.04	Expansion ≤ 0.04
Unsuitable for sulfate exposure	Expansion > 0.04	No requirement

**Table 15:** Sulfate resistance classification for concrete mixtures using accelerated method.

Mixture Name	28-Day Accelerated Expansion (%)	56-Day Accelerated Expansion (%)	Sulfate Resistance
C-T1	0.070	0.076	Unsuitable for sulfate exposure
C-T12	0.043	0.066	Unsuitable for sulfate exposure
C-T1-MK15	0.034	0.038	Suitable for moderate sulfate exposure
C-T12-MK15	0.015	0.016	Suitable for exposure to high sulfate concentrations

**Table 16:** Comparison between the concrete results from ASTM C1012-based method and accelerated method.

Mixture Name	Age at 0.04% Expansion (ASTM C1012-Based Method) (Day)	Sulfate Resistance (Accelerated Method)
C-T1	103	Unsuitable for sulfate exposure
C-T12	131	
C-T1-MK15	240	Suitable for moderate sulfate exposure
C-T12-MK15	277	Suitable for exposure to high sulfate concentrations

## Data Availability

The data presented in this study will be openly available in [Transportation Consortium of South-Central States], project No. 22CNMSU40 entitled “Accelerated Sulfate Attack Testing for Concrete”.
